# Estimating time since influenza virus exposure using single-cell proteomic data

**DOI:** 10.3389/fimmu.2026.1787198

**Published:** 2026-03-19

**Authors:** Klodiana Rizzo Nervo, Neda Hajiakhoond Bidoki, Han Chen, Zainab Rahil, Zach Bjornson, Kenneth Kim, Bonnie Bock, Melton Affrime, Logan Bauerle, Pham Bao Tran Huynh, David Liebowitz, Sean Tucker, Pier Federico Gherardini, Garry P. Nolan, Nima Aghaeepour, David R. McIlwain

**Affiliations:** 1 Department of Microbiology and Immunology, School of Medicine, University of Nevada, Reno, Reno, NV, United States; 2 Department of Anesthesiology, Perioperative and Pain Medicine, Stanford University School of Medicine, Stanford, CA, United States; 3 Department of Microbiology and Immunology, Stanford University School of Medicine, Stanford, CA, United States; 4 Ark Clinical Research, LLC, Long Beach, CA, United States; 5 WCCT Global, Cypress, CA, United States; 6 Vaxart, Inc., South San Francisco, CA, United States; 7 Department of Biology, University of Rome “Tor Vergata”, Rome, Italy; 8 Department of Pathology, Stanford University School of Medicine, Stanford, CA, United States

**Keywords:** controlled human virus challenge, influenza A (H1N1), machine learning, mass cytometry (CyTOF), random forest models

## Abstract

**Introduction:**

Determining when the onset of a respiratory infection occurred is important for effective clinical management and can aid in mapping transmission events. However, current diagnostic assays report only pathogen detection status and do not provide any information about the timing of infection, in part because of the lack of biomarkers that inform time since exposure.

**Methods:**

To address this gap, we developed immune-based predictive models of infection timing and shedding status using data from a controlled human challenge with influenza A/California/2009 (H1N1), in which major immune cell subsets were longitudinally profiled across multiple time points before and after viral challenge using 42-marker mass cytometry panels. Random forest machine learning models were trained to address two predictive objectives: (1) distinguishing virus shedders from non-shedders and (2) estimating days post-infection challenge (DPC) from immune profiles. Model performance was evaluated within the primary challenge cohort and independently validated using data from a separate controlled human influenza challenge study using the same virus.

**Results:**

Our analysis revealed that single-cell immune population dynamics alone encode a robust and reproducible temporal structure following influenza infection, enabling accurate estimation of virus exposure timing.

**Discussion:**

These findings provide foundational insight into host immune responses during influenza infection and represent an early step toward a future class of immune-based diagnostics that could extend beyond pathogen detection to inform infection timing and the duration of periods associated with contagiousness.

## Introduction

Influenza viruses pose a continual threat to public health by causing significant seasonal and pandemic respiratory diseases. In the United States alone, annual epidemics are estimated to cause 9–49 million infections, 140,000-710,000 hospitalizations, and 12,000-79,000 deaths each year (1). Worldwide, the catastrophic 1918 outbreak underscores the virus’s ability for rapid spread, severe morbidity, and large-scale mortality (2). Despite widespread vaccination efforts and the availability of antiviral therapies, influenza continues to exert a substantial and recurring health and economic burden.

Existing diagnostics for influenza and other respiratory infections rely primarily on rapid antigen detection assays and nucleic acid amplification tests (NAATs), such as reverse transcription-polymerase chain reaction (RT-PCR). Rapid antigen tests are inexpensive and offer near-immediate results but suffer from limited sensitivity. NAATs provide superior sensitivity and specificity but typically require more time, cost, and laboratory infrastructure (3). Clinically, both testing modalities generally offer only a binary readout of viral presence but reveal no information on when the infection began or how long an individual may remain infectious (4). As a result, clinicians and public authorities lack actionable tools to estimate infectious duration or guide time-sensitive decisions related to treatment, isolation, and contact tracing.

Controlled human challenge studies offer an ideal context to identify factors capable of estimating time since infection (TSI) because exposure timing is precisely defined. Prior efforts to estimate TSI have primarily focused on host transcriptional responses measured in controlled human challenge settings. Longitudinal gene expression signatures derived from influenza A (H1N1, H3N2), respiratory syncytial virus (RSV), and human rhinovirus (HRV) challenge studies have been shown to accurately infer timing of influenza exposure (5). Whole-blood transcriptomic analyses from individuals challenged with HRV, RSV, H1N1, and H3N2 have also identified early biomarkers that discriminate uninfected controls from individuals who subsequently become viral shedders within the first 32 hours post-infection (6). Additional studies have demonstrated distinct temporal transcriptional dynamics associated with asymptomatic versus symptomatic influenza A infection (7). Similar transcriptomic approaches applied to COVID-19 cohorts have successfully predicted both viral shedding status and stage of infection (8). Serological approaches have likewise been used to estimate infection recency beyond the acute phase (9).

While prior studies make it clear that peripheral blood contains temporal information, there remains a lack of studies leveraging single-cell data to estimate TSI for respiratory infections. Circulating cellular immune responses can capture coordinated changes across multiple immune compartments during infection and may provide a more direct path for identifying a minimal feature set needed for robust time since infection inference.

High-dimensional single-cell technologies, such as mass cytometry, also known as cytometry by time-of-flight (CyTOF), enable detailed characterization of immune system dynamics across infection stages by simultaneously measuring dozens of protein markers at the single-cell level. Mass cytometry has become a powerful tool to study infectious disease and vaccine immunology, offering the resolution needed to capture nuanced shifts in cellular immunity across multiple infection and vaccination contexts, including severe acute respiratory syndrome SARS-CoV-2, influenza A, dengue, Ebola, and others (10–16).

In this study, we analyzed peripheral blood samples from healthy adults challenged with influenza A/California/2009 (H1N1) using 42-marker mass cytometry panels to profile immune cell subsets before and after infection. We applied random forest machine learning models to classify subjects as virus shedders or non-shedders and to predict days post-infection challenge (DPC) from immune profiles, with model generalizability evaluated in an independent challenge cohort receiving the same influenza challenge virus. We demonstrate that high-dimensional single-cell immune population dynamics alone encode a robust and reproducible temporal signal following influenza infection, enabling accurate inference of infection timing across independent controlled human challenge studies. These results lay the groundwork for potential future diagnostic approaches capable of detecting infection and estimating when it began, and by extension, how long infectiousness may persist.

## Materials and methods

### Mass cytometry and clinical cohorts

Mass cytometry data were retrospectively analyzed from two independent controlled human influenza challenge studies, referred to as Study A and Study B, both of which used the same A/California/2009 (H1N1) challenge strain (17).

For Study A (11), data were analyzed from 35 volunteers with samples collected at baseline and on days 1–7 post-challenge (DPC). Volunteers were classified as non-shedders (n = 19) or viral shedders (n = 16), indicating having two or more positive nasopharyngeal swab (qRT-PCR) measurements of viral RNA.

For Study B (18), data were analyzed from 31 volunteers selected to provide a cohort comparable to Study A with respect to sampling timepoints and viral shedding status. This cohort included 15 non-shedders and16 shedders and, with representation across the three study treatment arms. Viral shedding status was determined in the original study using nasopharyngeal swab qRT-PCR data and was used as reported here. Samples were collected between days 1 and 7 DPC; however, the study design did not include collection at DPC4 or DPC6, and DPC7 samples were not collected for non-shedders or for four shedders.

Mass cytometry was performed on venipuncture whole blood samples that were fixed and cryopreserved using Smart Tube Proteomic Stabilizer. A consistent workflow for staining, data acquisition, and data processing was applied across both studies. Detailed descriptions of the experimental workflow and antibody staining panels used for each study have been published previously (11, 12).

Input data for machine learning consisted of mass cytometry measurements from the Study A and Study B cohorts. Collection day variables were standardized by converting study-specific day numbering schemes into a unified scale relative to viral challenge (DPC).

### Normalization

Cell population abundances from mass cytometry were calculated as frequencies of total mononuclear cells, as previously described (11, 12). To reduce noise associated with inter-individual variability, immune features were baseline-normalized by subtracting each participant’s pre-challenge values from post-challenge measurements. Two datasets were generated: baseline-normalized and not-normalized (raw). Both were analyzed using identical modeling pipelines, and model performance for viral shedding classification and time-since-infection prediction was compared to assess the impact of normalization.

### Classification model

For the binary classification task of distinguishing virus shedders from non-shedders, we implemented a random forest classifier (19). Within Study A, models were trained and validated both at individual DPC and across all days combined. Performance was evaluated using a 5-fold cross-validation strategy, in which folds were stratified by subject ID to ensure that all samples from a given participant were assigned exclusively to either the training or validation set. This design prevented information leakage across datasets and avoided artificially inflated performance estimates. The choice for five-folds was empirical and reflected a balance between computational efficiency and effective control over model bias and variance. To further strengthen robustness, the full cross-validation procedure was repeated one hundred times, and performance metrics were averaged across repetitions. Classification performance was summarized by the mean and standard deviation of key evaluation indicators, including receiver operating characteristics (ROC) and corresponding area under the curve (AUC) values. To ensure computational reproducibility, the random state of the random forest models was controlled using a fixed seed.

For external validation, models trained using Study A data were applied directly to the independent Study B cohort using the same feature set and preprocessing pipeline. No cross-validation was performed within Study B. Classification performance metrics were computed from predictions on Study B samples to enable direct comparison of model generalizability across cohorts.

### Receiver operating characteristics

Receiver Operating Characteristic (ROC) curves were generated to evaluate the performance of the random forest classifier in distinguishing virus shedders from non-shedders. ROC curves were constructed by plotting the true positive rate (sensitivity) against the false positive rate (1 – specificity) at varying classification thresholds. Sensitivity was calculated as TP/(TP + FN), and specificity as TN/(TN + FP). The area under the ROC curve (AUC) was computed as a scalar measure of the classifier’s discriminatory performance, ranging from 0 (no discrimination) to 1 (perfect discrimination).

ROC curves and AUC values were calculated using the scikit-learn Python library. Analyses were performed for each individual day post-infection as well as across all days combined. ROC/AUC metrics were also used to compare model performance with and without baseline normalization, allowing assessment of the contribution of normalization and immune feature dynamics to shedding classification.

### Regression model

To estimate the DPC at which each blood sample was collected, we trained a random forest regression model using immune cell population features from Study A. Model performance within Study A was evaluated using a leave-one-subject-out (LOSO) cross-validation strategy. In each fold, all samples from one participant across all available time points were held out, and predictions were generated for that participant’s samples using a model trained on all remaining participants. This approach ensured that each evaluation was performed on entirely unseen individuals, preventing data leakage and providing a realistic assessment of subject-level generalizability. Predictions from all LOSO folds were pooled to quantify overall model performance.

Model generalizability was then assessed using an independent external validation cohort (Study B). In this setting, the random forest model was trained once using all available samples from Study A and applied directly to Study B samples to generate predicted DPC values. No cross-validation was performed in Study B. Predicted DPC values were summarized as median trends with 95% confidence intervals. Model accuracy was quantified using Pearson correlation coefficients (r) and associated p-values to assess agreement between predicted and actual sampling days.

### Regression model prediction correlation

Pearson’s correlation coefficient (r) was used to assess the linear association between the regression model’s predicted values and the observed post-challenge time points. Correlation analyses were performed in Python using the pearsonr() function from the scipy.stats module, which provides both the correlation coefficient and a two-tailed p-value testing the null hypothesis of no correlation. Pearson correlation was used as a descriptive measure of agreement between predicted and observed DPC values to assess recovery of temporal structure along the infection timeline, rather than parametric assumption testing about the underlying data distributions.

### Root mean square error

Root Mean Square Error (rMSE) was used to quantify prediction accuracy in the random forest regression models. rMSE measures the average magnitude of prediction errors between the model’s predicted time post-infection and the actual time points of sample collection. rMSE was defined as follows, where *yi* are observed values and *ŷi* are predicted values:


$$rMSE=\sqrt{\frac{1}{n}\sum_{i=1}^{n}{\left(y_{i}-{\hat{y}}_{i}\right)}^{2}}$$

In this study, rMSE was calculated in two ways. First, rMSE was computed separately for each day post-challenge (DPC) using only samples collected at that time point, allowing prediction accuracy to be compared across stages of infection.

Second, an overall rMSE was computed by pooling all predictions across DPCs and calculating a single rMSE, equivalent to averaging the squared prediction errors across all samples before taking the square root. rMSE calculations were implemented in Python using NumPy by computing the square root of the mean squared difference between predicted and observed values, excluding non-finite entries.

### Correlation network analysis

Correlation networks were constructed to visualize univariate immune cell population feature importance and to contextualize these features based on cell population identity and similarity to other cell populations. Nodes represent gated immune cell populations, with node diameter encoding scaled univariate feature importance. Edges connect populations with similar phenotypic marker expression (Spearman ρ ≥ 0.7). Spearman correlation was used as a non-parametric measure to reduce sensitivity to non-normal distributions and outliers.

Nodes were embedded in two dimensions using t-distributed stochastic neighbor embedding (t-SNE) to cluster populations with similar phenotypic marker expression profiles. Nodes were also colored by cell population category. Univariate feature importance was quantified by the correlation between normalized population abundance and sampling day, summarized as -log10(p) or R² values, which were normalized for visualization and used to scale node diameter. Larger diameter nodes therefore indicate immune populations with relatively stronger infection- or time-associated patterns.

### Trajectory visualization by UMAP

Longitudinal immune cell trajectories were visualized using uniform manifold approximation and projection (UMAP) applied to immune cell frequency data across all samples and participants. Prior to dimensionality reduction, immune cell frequencies were normalized across samples to place features on a comparable scale. UMAP was then performed on the resulting feature matrix to embed samples into two dimensions, with UMAP1-UMAP2 coordinates reflecting similarity in immune cell frequency composition. Each sample was represented as a single point. The position of points in 2D space is dictated solely by immune cell frequency features and is independent of days post-challenge (DPC). After UMAP embedding, samples were colored by either actual or model-predicted DPC for visualization. The trajectory line traces the central tendency of samples across DPC for visualization purposes, with bold numbers marking each DPC.

### Software

All analyses were performed in Python using the pandas, NumPy, SciPy, Matplotlib, Seaborn, and scikit-learn libraries (20, 21), as commonly adopted in other single-cell studies (22).

## Results

### Study design

**Figure 1:**
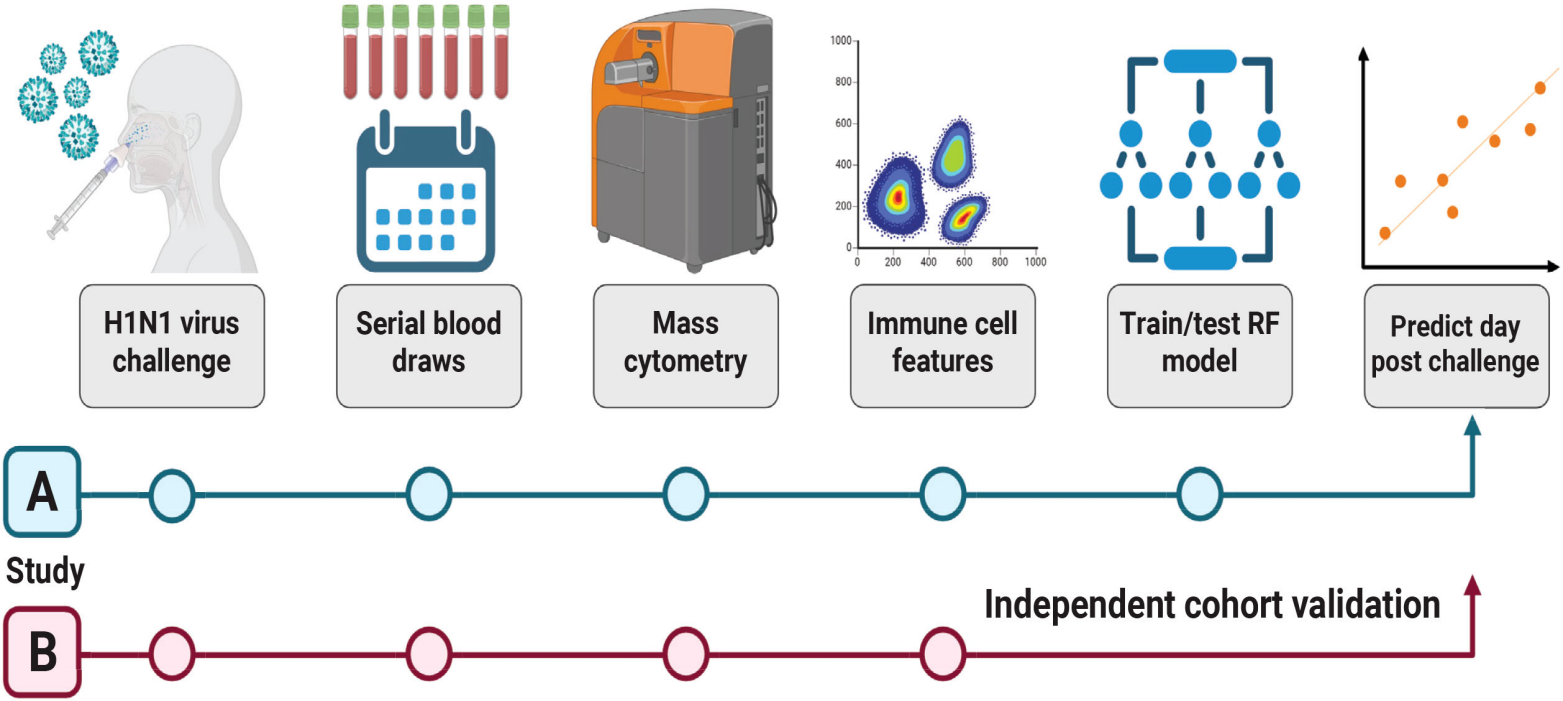
Study design and workflow for the prediction of virus shedding and time post-virus exposure. Blood samples were collected from volunteers before and after intranasal challenge with A/California/2009 (H1N1) virus as part of two independent studies. For Study A (11) and Study B (12), samples were analyzed from pre-challenge baseline days together with samples from up to 7 days post-challenge (DPC). Viral titers were measured by qRT-PCR in nasopharyngeal swabs to determine viral shedding status. For study A, virus challenge resulted in n=19 virus shedders and n=16 virus non-shedders. Study B samples were analyzed from n=16 shedders and n=15 non-shedders spread across treatment arms. Samples were analyzed by mass cytometry to identify immune cell subsets. Cell subset abundances were used as features to generate random forest machine learning models for the classification of viral shedding status and the prediction of DPC. Models built entirely using Study A train/test sets were subsequently independently validated using data from Study B.

The objective of this study was to determine whether integrated immune cell responses quantified by mass cytometry could be used in a machine learning framework to predict the timing of influenza virus infection. To achieve this, we analyzed data from two controlled human challenge studies using the same influenza A/California/2009 (H1N1) strain, described below. Use of an identical challenge strain and closely aligned experimental protocols across studies provided a consistent experimental context with respect to viral genetics and exposure conditions for evaluating temporal immune dynamics. Together, these cohorts provided complementary datasets for training and validating predictive models of infection dynamics (**Figure 1**).

Study A (11) enrolled 35 healthy adults who were intranasally inoculated with a standardized dose of A/California/2009 (H1N1), delivered by atomizer, and were intensively monitored during screening, confinement, and the post-challenge follow-up. Peripheral blood samples were collected daily until the seventh day post challenge (DPC), including two pre-challenge baseline samples. Participants were classified as virus shedders or non-shedders based on quantitative RT-PCR results from nasal swabs and supporting clinical assessments.

Study B (12, 18), was a clinical trial assessing the investigational oral influenza vaccine from Vaxart (VXA-A1.1) alongside a marketed vaccine and placebo control. These individuals were challenged with the same A/California/2009 (H1N1) strain under conditions aligned with study A. Peripheral blood samples were similarly collected across the course of infection, and shedder status was assigned using equivalent criteria applied in Study A.

### Immune profiling by mass cytometry

Mass cytometry was performed using 42-antibody panels to identify and quantify immune cell populations in peripheral blood samples collected from both studies. Manual gating resolved 37 immune cell populations (**Supplementary Table 1**), that reliably matched across both studies at consistent DPC and were used for modeling (**Supplementary Table 2**). These included major subsets of monocytes, granulocytes, B-cell, T-cell, and NK-cells. The full mass cytometry datasets from each study used as model inputs are provided in **Supplementary Table 3** and **Supplementary Table 4**. Prior analyses of the Study A dataset showed that these immune profiles resolved distinct immune response patterns between virus shedders and non-shedders (11).

**Figure 2:**
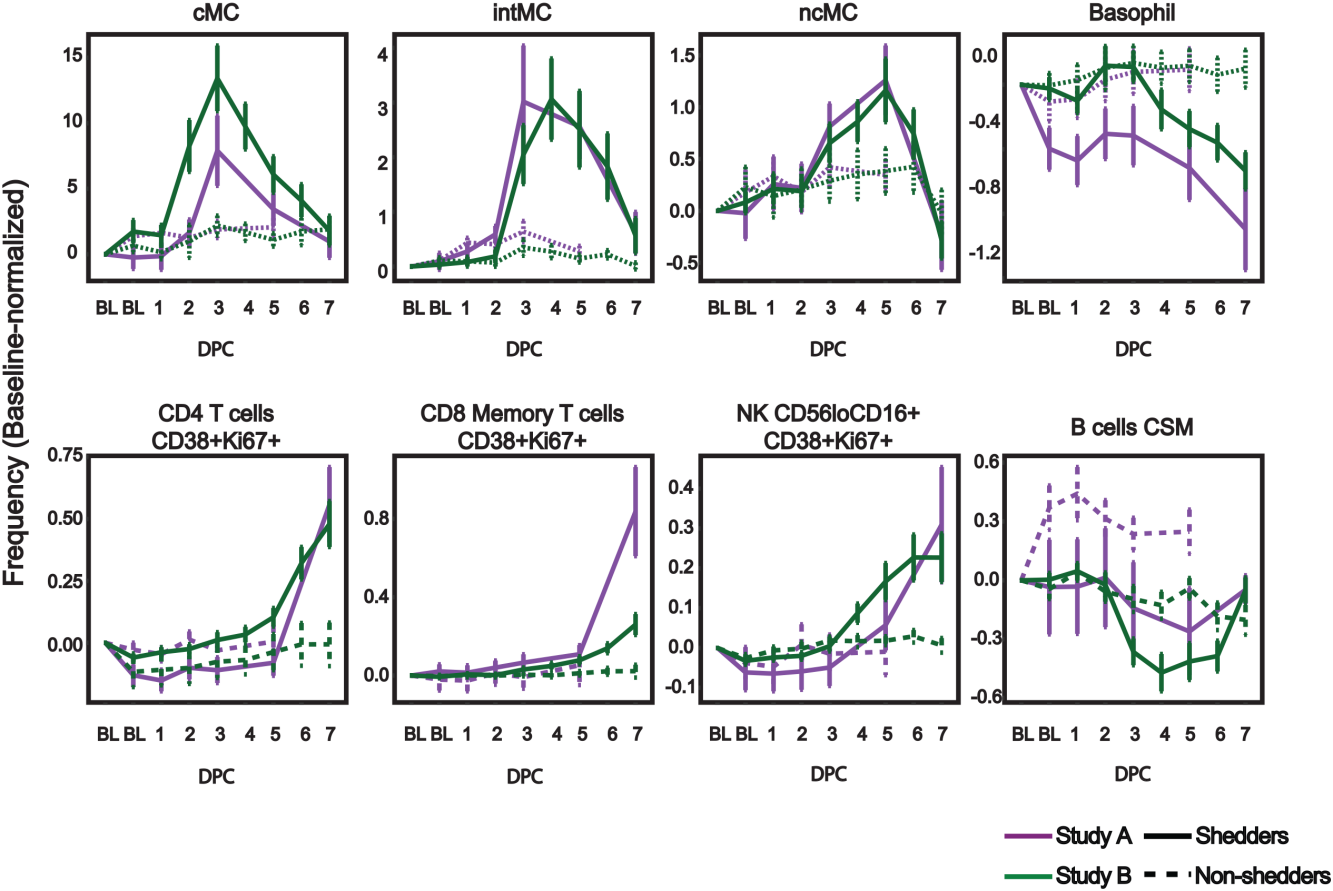
Post-challenge immune population kinetics in viral shedders align across studies. Relative abundances of indicated immune cell populations across baseline (BL) and day post-infection challenge (DPC). Classical monocytes (cMCs)(CD14+CD16-), intermediate monocytes (intMC)(CD14+CD16+), non-classical monocytes (ncMC)(CD16+CD14-), basophil (CD123+HLA-DR–), activated proliferating CD4 T cells (CD4+CD38+Ki67+), activated proliferating CD8+ Memory T cells (CD8+ CD45RA-CD38+ Ki67+), activated proliferating NK cells (CD56 loCD16+ CD38+ Ki67+), class-switched memory B cells (B cells CSM) (IgM-CD27+). For gating, see Rahil et al. and McIlwain and Chen et al. (11, 12). Values show relative abundance (% of mononuclear CD66- cells normalized by subtraction of baseline); filled squares and solid lines indicate virus shedders, and open squares and dashed lines indicate virus non-shedders. Green lines show samples from Study A (n=19 shedders, n=16 non-shedders), purple lines show samples from Study B (n=16 shedders, n=15 non-shedders). Plotted values are mean frequency +/- SEM.

Several immune populations displayed distinct population-specific temporal patterns following viral challenge (**Figure 2**). Classical monocytes (cMCs) and intermediate monocytes (intMCs) peaked around DPC 3 in shedders, with intMCs reaching maximum abundance slightly later on DPC 4. Nonclassical monocytes (ncMCs) increased until DPC 5 before declining. Basophils, defined as CD123^+^HLA-DR^−^ cells, decreased sharply in shedders. Activated and proliferating lymphocytes, including CD4^+^ T cells (CD38^+^Ki67^+^), CD8^+^ memory T cells (CD38^+^Ki67^+^), and NK cells (CD56^lo^CD16^+^ CD38^+^Ki67^+^), increased steadily in shedders through DPC 7. Class-switched memory B cells declined for shedders reaching nadirs at day 4 or 5. In contrast, non-shedders maintained relatively stable immune cell frequencies across most populations.

These coordinated and resolved changes in immune cell abundances over time reveal a clear trajectory of immune cell mobilization and contraction during influenza infection. The strong temporal structure of these immune responses suggests that peripheral immune profiles may encode sufficient information to infer the presence of infection and infection timing.

### Predicting viral shedding status with machine learning

To determine whether immune profiles could reliably distinguish infection outcomes, we trained random forest classifiers to differentiate virus shedders from non-shedders using baseline-normalized data. The models were trained and validated independently at each study day in both Study A and Study B. Furthermore, we used a bootstrapping strategy combined with five-fold cross-validation to account for the modest sample size and ensure robust performance estimates.

**Figure 3:**
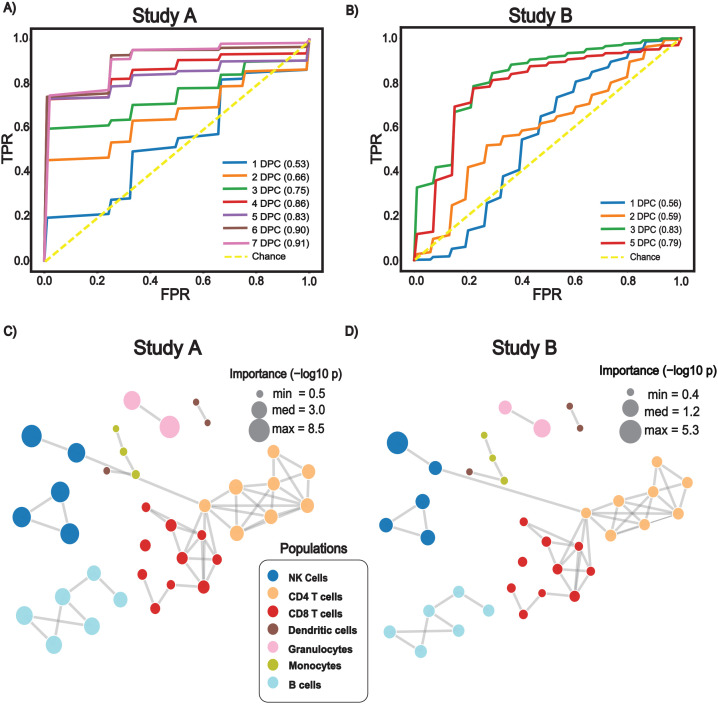
Random forest model effectively classifies virus shedding across studies. **(A, B)** Receiver operating characteristic (ROC) curves evaluating the performance of random forest models built using baseline-normalized data to classify virus shedders from non-shedders. Lines and AUC values show model performance at the indicated DPCs. **(A)** Model trained and validated on Study (A, B) Model trained on Study A and applied to the independent Study B cohort. **(C, D)** Correlation networks used to visualize and contextualize univariate immune population feature importance for Study A **(C)** and Study B **(D),** where nodes represent gated immune cell populations, and edges indicate similarity in phenotypic marker expression between populations. Univariate importance scores (–log10(p)) for distinguishing virus shedders from non-shedders are projected onto the network and scale node size, with larger nodes indicating stronger predictive importance. Networks contextualize the identity of immune cell populations and their relative importance to the classification models.

The classifiers achieved consistent predictive accuracy across multiple time points, with area-under-the-curve (AUC) values summarized in **Figures 3A, B**. Models trained on Study A data generalized to the independent Study B cohort with cross-cohort AUC values ranging from 0.50 to 0.81 across time points, indicating that peripheral immune signatures contain sufficient discriminatory power to classify viral shedding status following influenza challenge. In the absence of baseline normalization, models exhibited reduced overall predictive performance, indicating that anchoring immune features to each participant’s pre-challenge baseline is critical for amplifying infection-related immune signals (**Supplementary Figures 1A, B**).

To investigate the contribution of individual immune cell populations to model performance, we constructed network visualizations that highlight relationships among cell populations based on phenotypic marker expression (**Figures 3C, D**). Feature importance analyses indicated contributions from both innate and adaptive immune cell populations across studies, with NK cell and granulocyte subsets among the most consistently informative.

These results demonstrate that integrated immune cell population data provides a robust framework for distinguishing likely infection outcomes across independent human challenge studies.

### Predicting time since infection onset with machine learning

We next evaluated whether immune cell dynamics could be leveraged to infer the time since infection onset in viral shedders. Using Study A data, random forest regression models with one hundred estimators were trained using repeated stratified five-fold cross-validation (100 iterations), in which each iteration excluded one participant for testing while the model was trained on the remaining subjects. Both baseline-normalized and raw abundance data were analyzed to determine the effect of normalization on model performance, based on the expectation that incorporating each individual’s baseline immune profile would reduce biological variability and improve predictive accuracy.

**Figure 4:**
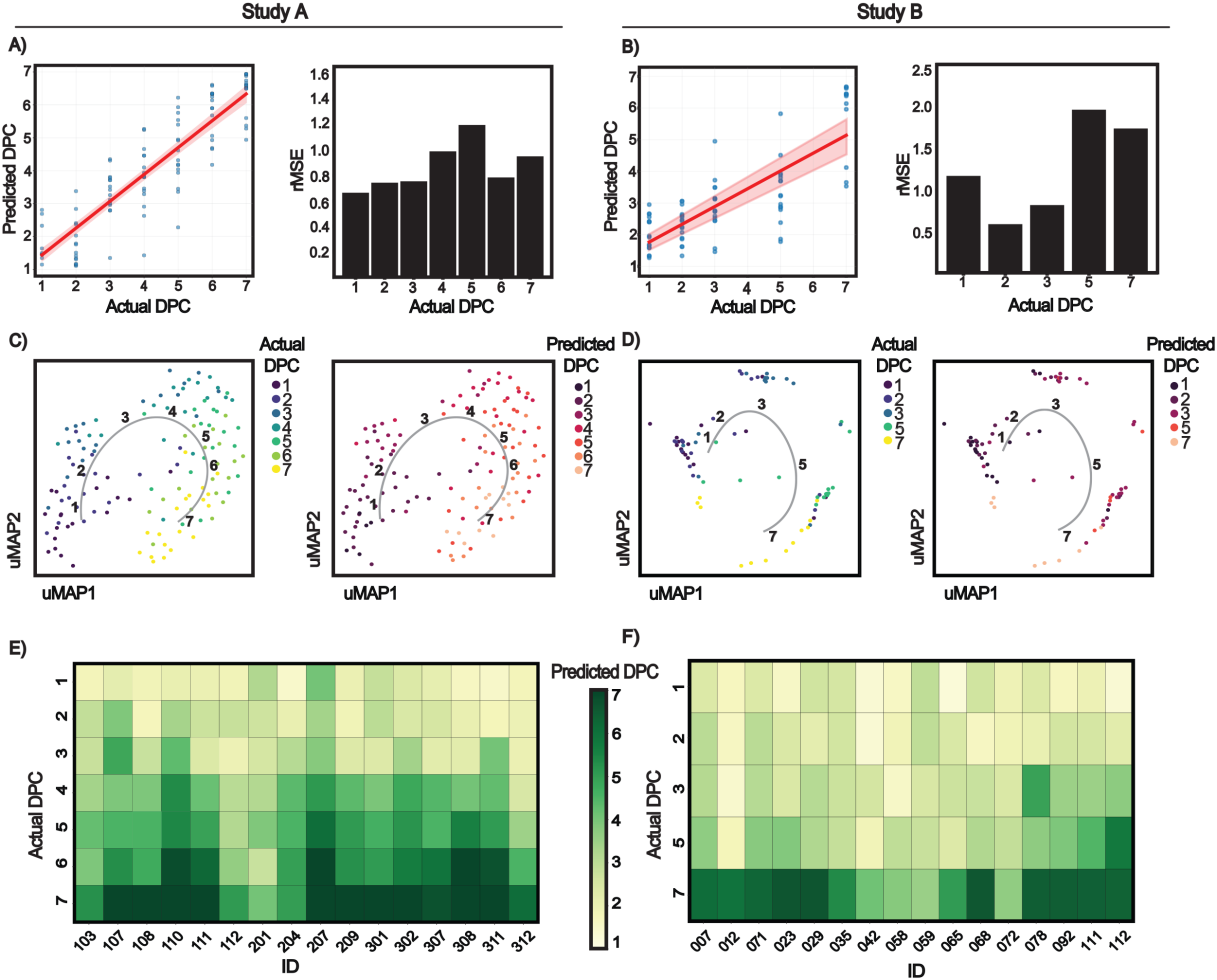
Machine learning–based prediction of time post virus exposure. **(A, B)** Random forest models predicting time post virus exposure in virus shedders using baseline-normalized data. **(A)** Leave-one-out cross-validation in Study A (Pearson r = 0.92, p = 5.87 × 10^−53^, n = 19) with corresponding root mean squared error (rMSE). **(B)** Model trained on Study A and applied to the independent Study B cohort (Pearson r = 0.78, p = 5.04 × 10^−17^, n = 16) with rMSE shown. **(C, D)** UMAP projections visualizing immune state trajectories over time and colored by actual or predicted DPC. UMAP projections of longitudinal immune cell frequencies from Study A **(C)** and Study B **(D)**, illustrating trajectories from baseline through peak infection and toward resolution; legends indicate whether color scale displays actual or model-predicted DPC, which show strong concordance. Each point represents a single sample embedded based on immune cell frequency features only (independent of DPC). Point colors are applied after UMAP embedding and do not influence the UMAP coordinates. The trajectory line traces the central tendency of samples across DPC, with bold numbers marking each DPC. **(E, F)** Heatmaps comparing predicted versus actual DPC by subject for Study A **(E)** and Study B **(F)**, demonstrating accurate temporal inference and generalization to an independent cohort.

For Study A, predicted DPC showed a strong correlation with actual DPC for viral shedders (r = 0.92, p-value = 5.87e^^−^53^ for normalized data; r = 0.70, p-value = 4.19e^-20^ for unnormalized data), as illustrated in **Figure 4A** and in **Supplementary Figures 2A, B**. Overall prediction error (rMSE, in days) was 1.060 for the Study A normalized model and 1.490 for the Study A unnormalized model. Predictions also generalized effectively by applying the model to the independent Study B cohort, yielding a robust correlation between predicted and actual DPC for viral shedders (r = 0.78, p-value = 5.04e^^−^17^ for normalized data; r = 0.57, p-value = 8.61e^-8^ for unnormalized data), as displayed in **Figure 4B** and in **Supplementary Figures 2C, D**. Corresponding overall rMSE values in Study B were 1.483 (normalized) and 1.490 (unnormalized). Attempts to model temporal trajectories in non-shedders did not produce meaningful temporal predictions, consistent with the absence of sustained infection-driven immune perturbation in these individuals (**Supplementary Figure 3**). Taken together, these results indicate that immune cell dynamics contain a measurable temporal signal that can be decoded to estimate time since infection, and that anchoring each participant’s data to their own baseline substantially enhances the stability and the accuracy of these estimates.

To examine the evolution of immune cell trajectories over the course of infection, we visualized all samples from all participants using uniform manifold approximation and projection (UMAP) built based on immune cell frequency data (**Figures 4C, D**). Here each point represents a sample, with UMAP1-UMAP2 coordinates reflecting global similarity in immune cell frequency composition. In both Studies A and B, samples followed an elliptical trajectory in UMAP space, with peak viral load timepoints located furthest from baseline. This pattern is consistent with a coordinated progression from infection onset through maximal immune activation and subsequent recovery. Points were colored by either actual or model-predicted days post-challenge (DPC). In both cases, samples with similar DPC clustered together along the UMAP trajectory, demonstrating close agreement between predicted timing and observed immune cell dynamics.

Model performance was further examined by comparing predicted versus actual DPC for each participant across each study (**Figures 4E, F**). The resulting heatmaps display accurate temporal ranking across individuals and timepoints. Equivalent heatmaps generated using unnormalized data showed reduced consistency, reinforcing the advantage of baseline-normalization (**Supplementary Figure 4**).

**Figure 5:**
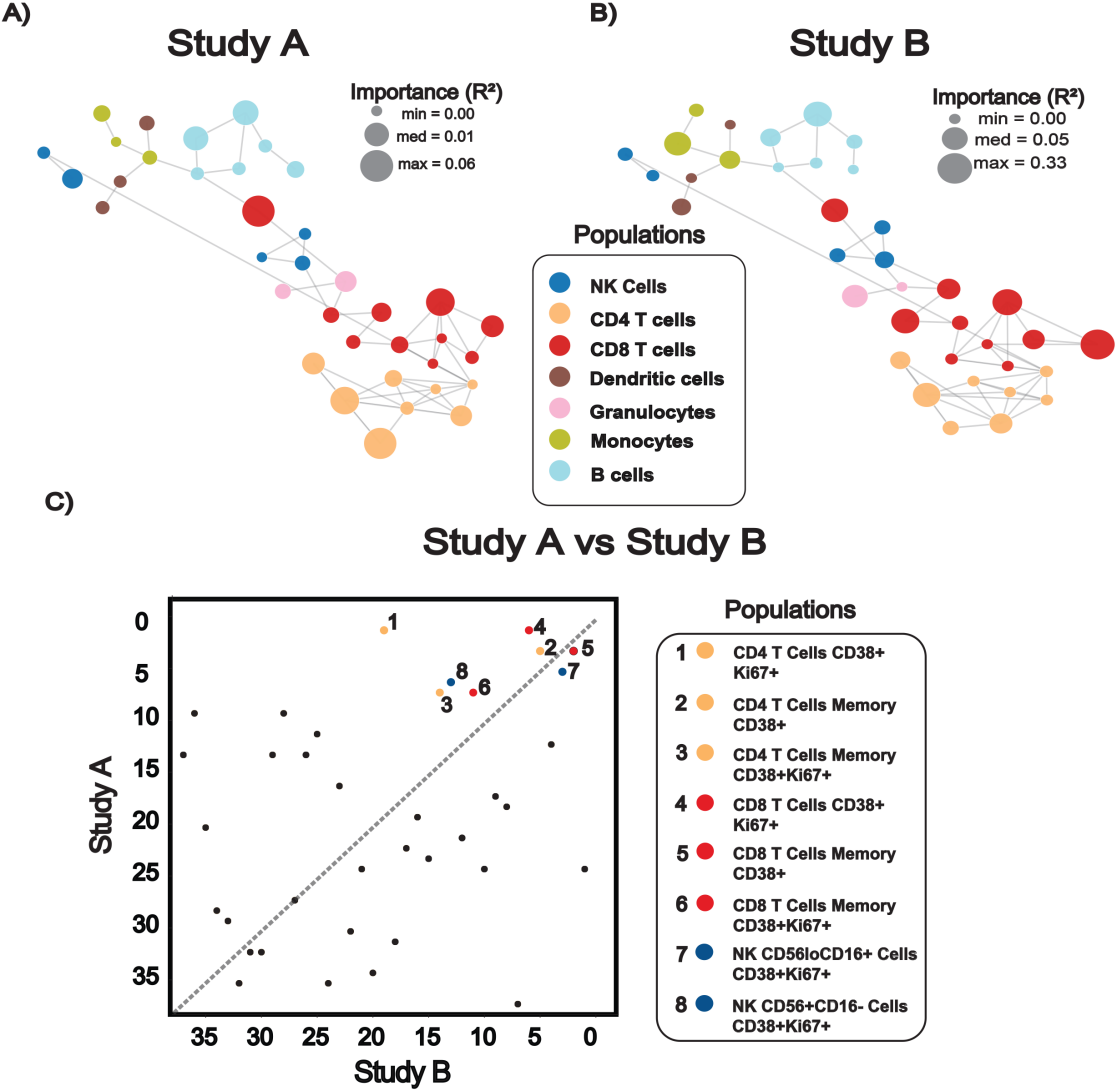
Network-based analysis of immune cell population importance for the prediction of time post virus exposure. **(A, B)** Correlation networks used to visualize and contextualize univariate immune population feature importance for Study A **(A)** and Study B **(B)**, where nodes represent gated immune cell populations, and edges indicate similarity in phenotypic marker expression between populations. Univariate importance scores (R²) for predicting DPC are projected onto the network and scale node size, with larger diameter nodes indicating stronger temporal predictive importance. Networks contextualize immune cell population identity and their relative importance to the predictive models. **(C)** Scatter plot for comparison of univariate model importance scores between Study A and Study B, where each point represents a cell population positioned according to its ranked importance in the two cohorts. Highly informative populations are highlighted, demonstrating consistent contributions of activated and proliferating T cell, T cell memory, and NK cells subsets to temporal prediction across studies.

Lastly, to explore cellular features driving temporal predictions, we applied the same network-based approach used for the classifier analyses. Networks depicting relationships among immune cell populations based on phenotypic marker expression were overlaid with univariate feature importance scores for days post-challenge (DPC) prediction (**Figures 5A, B**). Node size was scaled by the strength of each population’s association with time, as measured by Spearman correlation values, highlighting features with the strongest temporal relationships. To directly compare feature importance across studies, a scatter plot was used to visualize head-to-head rankings between models (**Figure 5C**). Immune populations consistently ranked among the most informative in both studies included activated and proliferating T cell and NK cell subsets (**Figure 5C**), consistent with their well-characterized expansion and activation dynamics during acute respiratory infection.

Collectively, these results suggest that temporal variations in immune cell populations encode a measurable and reproducible signal of infection timing, enabling reliable estimation of time since exposure across independent controlled human influenza challenge studies.

## Discussion

We applied machine learning to high-dimensional immune profiling data from two controlled human influenza challenge studies to demonstrate that circulating immune cell dynamics contain predictive information about infection stage. Immune population trajectories not only distinguished viral shedders from non-shedders but also enabled accurate prediction of time since infection challenge. Random forest models trained in one cohort generalized to an independent challenge study, underscoring the robustness and reproducibility of the temporal immune signatures identified under controlled experimental conditions. Together, these findings establish single-cell immune population dynamics as an independent and previously underexplored modality for infection staging.

Previous work has shown that respiratory viral infection stage can be inferred from blood-based biomarker data through transcriptional and serological profiling (5–9). Our results complement this body of work by demonstrating that single-cell immune phenotyping alone, without transcriptomic or serological data, captures temporal patterns of infection at high resolution. These findings indicate that cellular immune dynamics represent an underexplored source of biological information for infection staging. Future translation of these insights toward practical diagnostics will likely depend on identifying a minimal set of temporally informative cellular features that can be measured using scalable, lower-complexity platforms more suitable than mass cytometry.

Integrating measurement of such features with existing molecular diagnostic modalities could substantially improve clinical decision-making by providing estimates of infection status and timing, enabling more precise guidance on treatment interventions. Because the temporal relationship between time since exposure and viral shedding has been characterized for influenza, accurate estimation of time since infection alone may provide an indirect framework to improve estimates of expected shedding windows. Whether similar immune-cell–based models could be developed in studies with larger cohorts and more precisely resolved longitudinal shedding measurements to directly predict the duration of viral shedding warrants investigation in future work. Identifying circulating immune cell responses predictive of atypical prolonged viral shedding in community-acquired respiratory viral infection would be particularly valuable.

In this study, model performance benefited from normalization to individual pre-challenge immune baselines, highlighting a dependency on baseline measurements for optimal temporal inference. Although this requirement could pose practical challenges for deployment, it also underscores the broader value of establishing individual immune baselines for interpreting immune perturbations, which may prove relevant across multiple healthcare contexts, including infection, vaccination, and other inflammatory or physiological stressors (23). As longitudinal immune profiling becomes more feasible, baseline-referenced models may support more precise interpretation of immune state changes.

Influenza A viruses evolve through antigenic drift and, less frequently, antigenic shift, with prominent variation in the surface glycoproteins hemagglutinin (HA) and neuraminidase (NA). Viral genetic differences can alter replication kinetics, host immunity, and the timing and magnitude of inflammatory and cellular responses (2). An important limitation of our study is that models were trained and validated using two studies conducted with a single A/California/2009 (H1N1) challenge strain; the extent to which the same immune features and learned mappings generalize to antigenically distinct strains remains uncertain. Contemporary influenza A controlled human infection models have largely focused on H1N1 and H3N2 challenge strains (24), reflecting practical and ethical constraints on human challenge-strain development and use. An important next step is to evaluate performance across additional controlled influenza challenge strains; matched multi-strain challenge cohorts would enable a direct test of model generalizability.

Furthermore, because this analysis was performed in controlled human influenza challenge studies with precisely defined exposure timing and standardized infection conditions, additional work would be required to evaluate how immune-cell-based temporal inference models perform in community-acquired influenza settings. In natural infection contexts, greater variability in viral genetics, infectious dose, and host population characteristics may influence immune response trajectories, while less uniform clinical sampling schedules may introduce additional noise. Cohort sizes in the present study were modest, constraining model complexity and limiting power to detect subtler cellular features. Future studies in larger and more diverse populations that span broader influenza strain diversity and reflect real-world, less uniform community sampling will be essential to evaluate generalizability and to develop more comprehensive temporal prediction models that remain robust to evolving influenza viruses.

In summary, this study demonstrates that coordinated changes in circulating immune cell populations encode measurable information about infection timing that can be extracted using high-dimensional phenotyping coupled with machine learning. Across two independent controlled human influenza challenge studies, these immune dynamics supported accurate inference of infection stage and generalized across cohorts challenged with the same viral strain. Together, these findings establish a benchmark for immune-based temporal inference under well-defined experimental conditions and provide a foundation for future studies evaluating the performance of such approaches in more heterogeneous, community-acquired infection settings.

## Data Availability

The datasets used for model building in this study are available in **Supplementary Tables 3** and **4**. Code used in this study is available from Github (https://github.com/davemcilwain/Flu-Day-Prediction).

## Supplementary material

Supplementary Figure 1 Random forest model classifying virus shedding across studies without baseline normalization. ROC curves evaluating the performance of random forest models built using raw, not-normalized data to classify virus shedders from non-shedders. Lines and AUC values show model performance at the indicated DPCs. **(A)** Model trained and validated on Study A. **(B)** Model trained on Study A and applied to the independent Study B cohort. Results illustrate reduced overall classification accuracy and generalization compared to baseline-normalized models shown in **Figure 3**.

Supplementary Figure 2 Machine learning–based prediction of time post virus exposure without baseline normalization. **(A–D)** Random forest models predicting time post virus exposure in virus shedders using raw, not-normalized data. **(A, B)** Leave-one-out cross-validation in Study A (Pearson r = 0.70, p = 4.19 × 10^−20^, n = 19) with corresponding root mean squared error (rMSE). **(C, D)** Model trained on Study A and applied to the independent Study B cohort (Pearson r = 0.57, p = 8.61 × 10^−^^8^, n = 16) with rMSE shown. Results illustrate reduced temporal accuracy and generalization compared to baseline-normalized models shown in **Figure 4**.

Supplementary Figure 3 Expected absence of predictive temporal immune trajectories in non-shedders. Random forest models predicting time post virus exposure in non-shedders. **(A–D)** Models built using baseline-normalized data. **(A)** Leave-one-out cross-validation in Study A (Pearson r = 0.45, p = 1.26 × 10^−7^, n = 16) with corresponding root mean squared error (rMSE) shown in **(B)**. **(C)** Model trained on Study A and applied to the independent Study B cohort (Pearson r = 0.17, p = 1.93 × 10^−^¹, n = 15) with rMSE shown in **(D)**. **(E–H)** Models built using raw, not-normalized data. **(E)** Leave-one-out cross-validation in Study A (Pearson r = 0.27, p = 2.13 × 10^−^³, n = 16) with rMSE shown in **(F)**. **(G)** Model trained on Study A and applied to the independent Study B cohort (Pearson r = 0.01, p = 9.14 × 10^−^¹, n = 15) with rMSE shown in **(H)**.

Supplementary Figure 4 Predicted versus actual DPC without baseline normalization. **(A, B**) Heatmaps comparing predicted versus actual DPC by subject for Study A **(A)** and Study B **(B)** using raw, non-normalized data. Each tile represents the median predicted DPC for a given subject relative to the actual DPC, illustrating reduced temporal accuracy and generalization compared to baseline-normalized models shown in **Figure 4**.

Supplementary Table 1 Mapping of immune cell populations between study A and study B. Columns list cell populations from each study dataset, with corresponding or equivalent subpopulations aligned within the same row.

Supplementary Table 2 Alignment of sampling timepoints between study A and study B. The DPC (days post-challenge) column indicates the unified timepoint alignment used in this study. The Study A and Study B columns list the original study-specific day numbering schemes reported in (11) and (12) respectively. For Study A, viral challenge occurred on study day 1 (original numbering) following blood draw sampling. For Study B, viral challenge occurred on study day 90 (original numbering) following blood draw sampling. For both studies, the DPC 1 blood draw corresponds to approximately 24 hours post-challenge.

Supplementary Table 3 Study A immune cell abundance data used for model features. Columns include volunteer identifier (VOLUNTEER), day post-challenge of blood draw (DPC), and virus shedding status (SHEDDER), encoded as 1 for shedders and 0 for non-shedders, for participants in Study A. Remaining columns report the relative abundances of immune cell populations measured by mass cytometry and used as model features.

Supplementary Table 4 Study B immune cell abundance data used for model features. Columns include volunteer identifier (VOLUNTEER), day post-challenge of blood draw (DPC), and virus shedding status (SHEDDER), encoded as 1 for shedders and 0 for non-shedders, for participants in Study B. Remaining columns report the relative abundances of immune cell populations measured by mass cytometry and used as model features.
