# Supplementary material for: Estimating time since influenza virus exposure using single-cell proteomic data
Source: Front Immunol. 2026 Mar 19;17:1787198. doi: 10.3389/fimmu.2026.1787198 (PMC13044454; doi:10.3389/fimmu.2026.1787198)
Supplement: Supplementary Figure 1 — Random forest model classifying virus shedding across studies without baseline normalization. ROC curves evaluating the performance of random forest models built using raw, not-normalized data to classify virus shedders from non-shedders. Lines and AUC values show model performance at the indicated DPCs. (A) Model trained and validated on Study A. (B) Model trained on Study A and applied to the independent Study B cohort. Results illustrate reduced overall classification accuracy and generalization compared to baseline-normalized models shown in Figure 3. [file Presentation1.pdf]

Supplementary Materials

Estimating time since influenza virus exposure using single-cell proteomic data.

Rizzo Nervo K, Hajiakhoond Bidoki N, Chen H, Rahil Z, Bjornson-Hooper Z, Kenneth K, Bock B, Affrime M, Bauerle L, Huyn PBT, Liebowitz D, Tucker S, Gherardini PF, Nolan G, Aghaeepour N, McIlwain DR.

Front Immunol. 2026;17. doi:10.3389/fimmu.2026.1787198

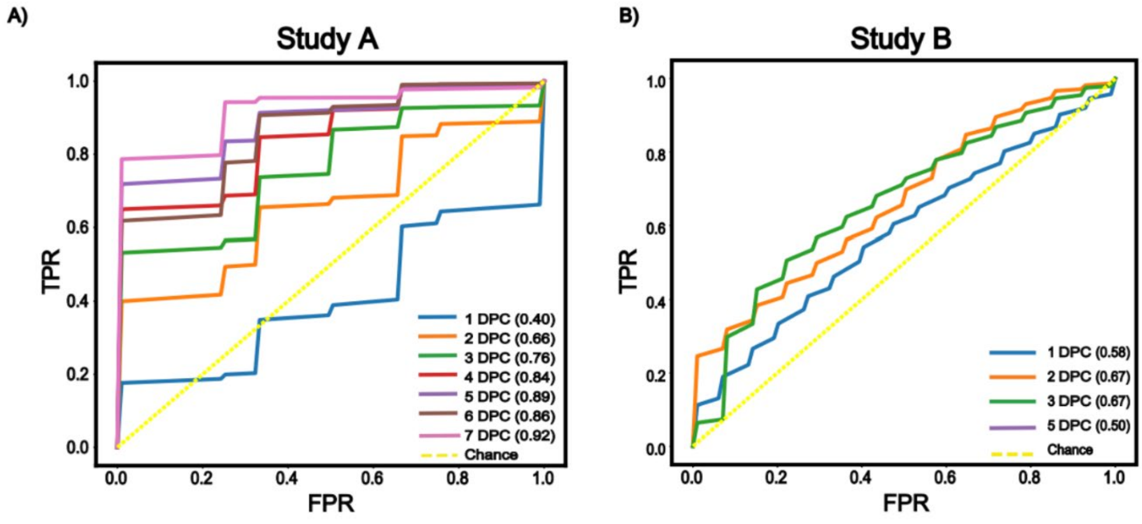

Supplementary Figure 1. Random forest model classifying virus shedding across studies without baseline normalization. ROC curves evaluating the performance of random forest models built using raw, not-normalized data to classify virus shedders from non-shedders. Lines and AUC values show model performance at the indicated DPCs. **(A)** Model trained and validated on Study A. **(B)** Model trained on Study A and applied to the independent Study B cohort. Results illustrate reduced overall classification accuracy and generalization compared to baseline-normalized models shown in Figure 3.

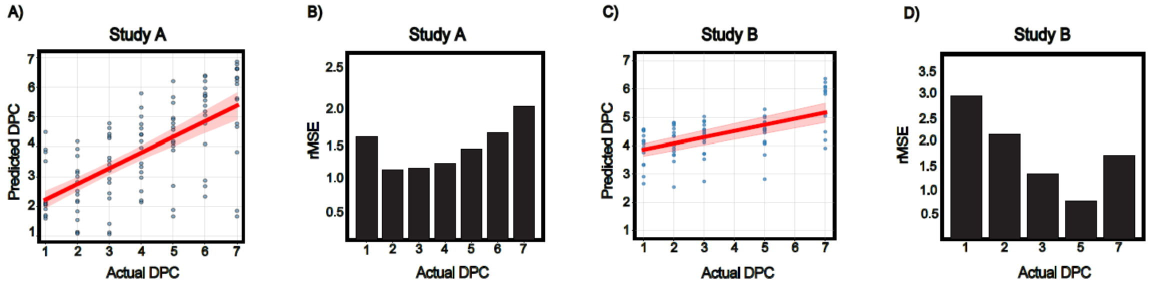

Supplementary Figure 2. Machine learning–based prediction of time post virus exposure without baseline normalization. (A-D) Random forest models predicting time post virus exposure in virus shedders using raw, not-normalized data. **(A,B)** Leave-one-out cross-validation in Study A (Pearson $r = 0.70$, $p = 4.19 \times 10^{-20}$, $n = 19$) with corresponding root mean squared error (rMSE). **(C,D)** Model trained on Study A and applied to the independent Study B cohort (Pearson $r = 0.57$, $p = 8.61 \times 10^{-8}$, $n = 16$) with rMSE shown. Results illustrate reduced temporal accuracy and generalization compared to baseline-normalized models shown in Figure 4.

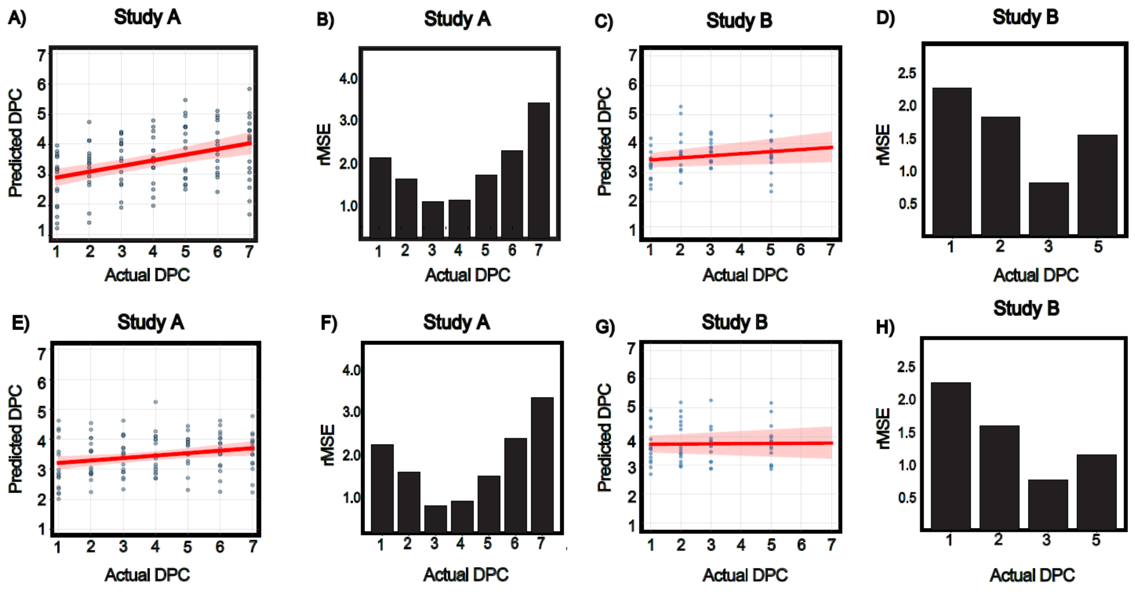

Supplementary Figure 3. Expected absence of predictive temporal immune trajectories in non-shedders. Random forest models predicting time post virus exposure in non-shedders. **(A–D)** Models built using baseline-normalized data. **(A)** Leave-one-out cross-validation in Study A (Pearson r = 0.45, p = 1.26×10^{-7}, n = 16) with corresponding root mean squared error (rMSE) shown in **(B)**. **(C)** Model trained on Study A and applied to the independent Study B cohort (Pearson r = 0.17, p = 1.93×10^{-1}, n = 15) with rMSE shown in **(D)**. **(E–H)** Models built using raw, not-normalized data. **(E)** Leave-one-out cross-validation in Study A (Pearson r = 0.27, p = 2.13×10^{-3}, n = 16) with rMSE shown in **(F)**. **(G)** Model trained on Study A and applied to the independent Study B cohort (Pearson r = 0.01, p = 9.14×10^{-1}, n = 15) with rMSE shown in **(H)**.

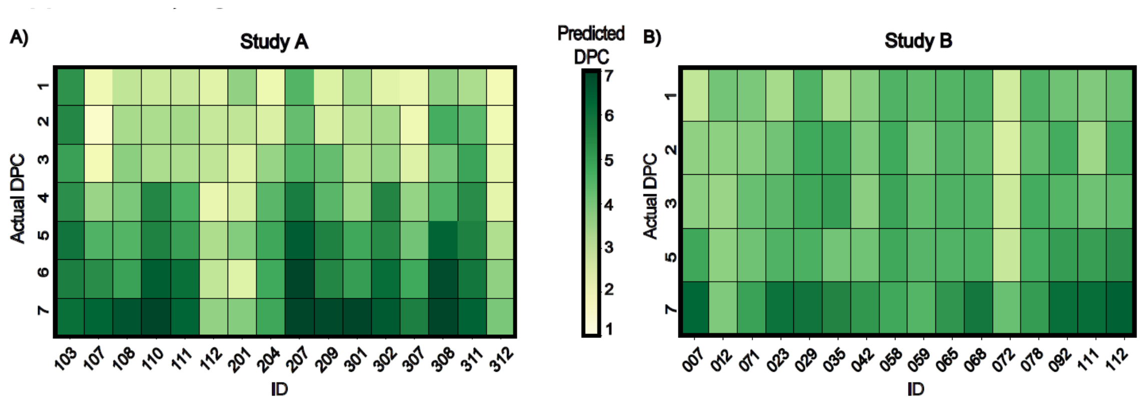

Supplementary Figure 4. Predicted versus actual DPC without baseline normalization. **(A, B)** Heatmaps comparing predicted versus actual DPC by subject for Study A **(A)** and Study B **(B)** using raw, non-normalized data. Each tile represents the median predicted DPC for a given subject relative to the actual DPC, illustrating reduced temporal accuracy and generalization compared to baseline-normalized models shown in Figure 4.

[Data Sheet 1.xlsx]

Supplementary Table 1. Mapping of immune cell populations between Study A and Study B. Columns list cell populations from each study dataset, with corresponding or equivalent subpopulations aligned within the same row.

[Data Sheet 2.xlsx]

Supplementary Table 2. Alignment of sampling timepoints between Study A and Study B. The DPC (days post-challenge) column indicates the unified timepoint alignment used in this study. The Study A and Study B columns list the original study-specific day numbering schemes reported in (11) and (12) respectively. For Study A, viral challenge occurred on study day 1 (original numbering) following blood draw sampling. For Study B, viral challenge occurred on study day 90 (original numbering) following blood draw sampling. For both studies, the DPC 1 blood draw corresponds to approximately 24 hours post-challenge.

[Data Sheet 3.xlsx]

Supplementary Table 3 . Study A immune cell abundance data used for model features. Columns include volunteer identifier (VOLUNTEER), day post-challenge of blood draw (DPC), and virus shedding status (SHEDDER), encoded as 1 for shedders and 0 for non-shedders, for participants in Study A. Remaining columns report the relative abundances of immune cell populations measured by mass cytometry and used as model features.

[Data Sheet 4.xlsx]

Supplementary Table 4. Study B immune cell abundance data used for model features. Columns include volunteer identifier (VOLUNTEER), day post-challenge of blood draw (DPC), and virus shedding status (SHEDDER), encoded as 1 for shedders and 0 for non-shedders, for participants in Study B. Remaining columns report the relative abundances of immune cell populations measured by mass cytometry and used as model features.
